# Exploring the Experiences and Needs of Patients With Type 2 Diabetes Mellitus in Sleman Regency, Yogyakarta, Indonesia: Protocol for a Qualitative Study

**DOI:** 10.2196/37528

**Published:** 2022-09-06

**Authors:** Yunita Linawati, Erna Kristin, Yayi Suryo Prabandari, Susi Ari Kristina

**Affiliations:** 1 Faculty of Medicine, Public Health and Nursing Gadjah Mada University Yogyakarta Indonesia; 2 Department of Pharmacology Faculty of Medicine, Public Health and Nursing Gadjah Mada University Yogyakarta Indonesia; 3 Department of Health Behavior, Environment Health & Social Medicine Faculty of Medicine, Public Health and Nursing Gadjah Mada University Yogyakarta Indonesia; 4 Department of Pharmaceutics Faculty of Pharmacy Gadjah Mada University Yogyakarta Indonesia

**Keywords:** type 2 diabetes mellitus, social cognitive theory, personal factors, environment, behavior, knowledge, attitude, adherence, HbA1c, hemoglobin A1c

## Abstract

**Background:**

Type 2 diabetes mellitus (T2DM) is a chronic disease that can cause adverse effects if not managed effectively. The prevalence of T2DM will continue to rise every year, and data from the International Diabetes Federation show that the number of patients diagnosed with T2DM in Indonesia is predicted to increase from 10.3 million in 2017 to 16.7 million in 2045. Managing T2DM properly is a challenge for the patients because they need to implement lifestyle changes that involve the self-monitoring of blood glucose, consuming prescribed medication properly, maintaining a healthy diet, getting sufficient physical training, keeping a healthy sleeping pattern, managing stress properly, and consulting medical professionals regularly. The worldwide intervention for T2DM focuses on self-management education. The varied results in studies about interventions show that no particular intervention method can be regarded as the most effective. In Indonesia, there are limited studies on educational interventions to improve the quality of life and health of patients with T2DM.

**Objective:**

This study aims to explore the experiences and needs of patients with T2DM in Sleman Regency, Yogyakarta, Indonesia, to develop effective self-management education.

**Methods:**

The study will use the phenomenology method with purposive sampling to collect data. The inclusion criteria are patients in the Chronic Disease Self-Management Program at the Sleman Regency Public Health Center who are aged ≥18 years, diagnosed with T2DM for more than a year, with hemoglobin A1c levels ≤7.5% and >7.5%, capable of communicating verbally and literate in the Indonesian language, not deaf, and willing to participate. The data collection is based on the Social Cognitive Theory, which involves selecting assessment targets and analyzing personal factors, environment, and behavior that determine the knowledge, attitude, and adherence of persons with T2DM. Researchers will collect the data through in-depth, face-to-face interviews to learn about knowledge, self-efficacy, outcome expectancy, outcome experience, worry, illness belief, treatment belief, diet, physical activity, medicine intake, treatment pattern, support system, as well as ethnic and cultural influences. The results will be taken from unstructured and open-ended questions written in Indonesian according to the interview guidelines. The data analysis process will go through several stages: reading the data thoroughly; coding; sorting the categories; creating the themes; making general descriptions; and presenting the data in charts, narratives, and recorded quotations from the interviews.

**Results:**

This study received a grant in May 2021 and gained permission from the Medical and Health Research Ethics Committee of Universitas Gadjah Mada, Indonesia, on July 1, 2021. Data collection started on August 12, 2021, and the results are expected to be published in 2022.

**Conclusions:**

The results of this study will be used to design an educational intervention model to improve the knowledge, attitude, and adherence of patients with T2DM.

**International Registered Report Identifier (IRRID):**

DERR1-10.2196/37528

## Introduction

### Background

The prevalence of type 2 diabetes mellitus (T2DM) worldwide is projected to increase from 463 million in 2019 to 700.2 million in 2045 [1]. This global trend makes diabetes an epidemic [2,3]. Indonesia has the 7th largest number of patients with T2DM with 10 million confirmed cases and 5.2 million undiagnosed cases [1]. T2DM contributes to 2% of the mortality rate worldwide and 6% of the mortality rate in Indonesia [4,5]. Globally in 2019, T2DM and its complications caused the deaths of around 4.2 million people aged 20-79 years. At this rate, it means that every 8 seconds, 1 person dies of T2DM, with nearly half (46.2%) of the mortality coming from the working-age population (aged <60 years). The increased mortality rate and decreased quality of life caused by T2DM and its complications affect the health and economy of the patient, family, society, and even the country’s health system [1].

Managing T2DM properly is a challenge for the patients because they need to implement lifestyle changes that involve the self-monitoring of blood glucose, consuming prescribed medication properly, maintaining a healthy diet, getting sufficient physical training, keeping a healthy sleeping pattern, managing stress properly, and consulting medical professionals regularly [6]. Various problems faced by patients with T2DM include an unhealthy diet, the lack of physical activity, and poor knowledge about how the disease and treatment affect the patients’ treatment and medication adherence [7]. Several factors that affect medication adherence among patients with T2DM are therapy duration; complex treatment; miscommunication between patients and medical staff; the lack of information; psychological factors; and the patients’ poor perception of the benefits, security, side effects, and cost of the treatment [8,9]. The lack of adherence will increase the risk of microvascular and macrovascular complications [7,10-14]. Patient adherence to treatment is the key to successfully treating chronic diseases such as T2DM [15-18]. The lack of knowledge regarding the treatment also corresponds to poor patient adherence and causes treatment failure [19].

Another problem related to T2DM in Indonesia involves the public misperceptions about the disease and treatment. One study found several public false perceptions about T2DM [20]. Another study says that only people of high economic status can have T2DM and that people of low economic status cannot possibly have the disease [20]. People also believe that T2DM is a hereditary disease that only affects those with a family history of T2DM. Furthermore, people still believe that they could prevent T2DM if they live a traditional lifestyle and deny that an unhealthy diet and smoking can cause T2DM [20]. Some patients think that T2DM is not a serious disease because they can still do daily activities. According to some people’s religious belief, T2DM is given to them by God [21]. T2DM is also considered to be bad karma and inherited from a past life as a hereditary disease. Many patients with T2DM experience stress because they cannot accept their condition [22]. Many patients have low medication adherence and prefer alternative or complementary therapy [23]. Additionally, traditional medication has become popular because people have more trust in traditional medical practitioners than modern medical practices. Additionally, they are worried about the side effects of modern medication and remain skeptical about its efficacy [24].

### Social Cognitive Theory

We will use Bandura’s Social Cognitive Theory (SCT) as the basis of this qualitative study plan. According to the theory, the 3 components that affect someone to change their health behavior are self-efficacy, purpose or intention, and outcome expectancy [25]. Bandura expounded human behavior in terms of triadic reciprocal causation, which refers to the causal relation of personal and cognitive factors, behavior, and environment. These 3 factors work interdependently as the defining factors with other bioecological aspects [26]. Bandura’s SCT emphasized the strong connection between personal factors, behavior, and environment, supported by learning through observation [27,28]. The main elements are knowledge, social support, outcome expectancy, self-regulation, and self-efficacy [28]. SCT is highly effective in predicting and explaining patient adherence to diabetes treatment [28]. Self-efficacy is closely related to the individuals’ belief in their capacity to perform certain behaviors, such as exercising [29]. However, most researchers only focus on some SCT components, namely self-efficacy and self-regulation, and ignore the other aspects [28]. One study shows that intervention on patients with T2DM using SCT can improve patient adherence to proper diet, physical activity, treatment, self-foot check, and the self-monitoring of blood glucose [30]. Another study found a similar result, showing that the intervention model using SCT could also increase physical activity and adherence to prescribed medication [31]. A systematic review revealed that the interventions using SCT on patients with T2DM can increase patient adherence to most aspects of T2DM treatment with a total quality score of 9 out of 10 and shows a more consistent result than the interventions using other behavioral theories [32]. SCT is considered the most effective model to improve patient adherence to most aspects of T2DM treatment [30,31] and continues to be one of the theories that can be applied to arrange a comprehensive model to change behavior [28].

### Aim and Research Questions

This study will explore the experiences and needs of patients with T2DM in treating T2DM by analyzing personal factors, environment, and behavior.

The following 2 questions will be addressed:

1. What are the experiences of patients with T2DM in managing their disease?
2. What are the needs of patients with T2DM in managing their disease?

## Methods

### Study Approach

The study will use the phenomenology approach to describe human experiences about a certain phenomenon [32]. We chose phenomenology because this approach is a research strategy used to identify the nature of human experiences regarding the meaning of life as perceived by patients with T2DM. Understanding human experiences makes phenomenology a research method with procedures that require the researchers to analyze the subjects by getting involved directly for a relatively long time to develop the patterns and connections of meaning [32,33]. In this process, the researchers must set personal experiences aside to understand the experiences of patients with T2DM in bringing meaning to their lives. We intend to gain a complete understanding of the patients’ knowledge, attitude, and adherence and the barriers they encounter.

### Participants

The study participants will be patients in the Chronic Disease Self-Management Program at the Sleman Regency Public Health Center, Yogyakarta, Indonesia, who are aged ≥18 years, diagnosed with T2DM for more than a year, receiving oral medication therapy and not using insulin, have hemoglobin A_1c_ levels ≤7.5% and >7.5%, capable of communicating verbally and literate in the Indonesian language, not deaf, and willing to participate in the research.

### Participant Recruitment

The participants will be identified according to the data provided by the Sleman Regency Public Health Center after obtaining the research permit. Subsequently, the participants will be selected according to the criteria stated above. We will hold activities in collaboration with the public health center and public health cadres. Public health cadres are community members who are selected and trained by the community health center to improve public health. Through these cadres, the candidates (patients in the Chronic Disease Self-Management Program diagnosed with T2DM) will be identified. Once all of the requirements are fulfilled, we will choose the candidates to participate in the in-depth interviews.

### Sampling

Qualitative research focuses on depth and process; hence, only a relatively small number of participants (less than 10 people) will be examined in this phenomenological study. The participants will be selected using the purposive sampling technique. The sample will be considered sufficient once the obtained information reaches data saturation or data satisfaction, which refers to the point where no new information can be discovered from additional participants.

### Participant Compensation

Upon the completion of the procedure, each participant will receive Rp 100,000.00 (US $6.77) as a token of appreciation for participating in the research.

### Interview Procedure and Data Collection

The data collection components in this research will be interviewers (researchers and field assistants), interview guidelines, field notes, a recorder, and a camera. The research guideline for interviewing patients with T2DM will emphasize the following aspects: knowledge, self-efficacy, outcome expectancy, outcome experience, worry, illness belief, treatment belief, diet, physical activity, medicine intake, treatment pattern, support system, as well as ethnic and cultural influences. Data collection will be done through in-depth interviews, which requires conducting a face-to-face meeting. This type of interview uses unstructured and open-ended questions compiled according to the guidelines to gain insights and opinions from the participants. The time allocated for each participant in 1 meeting will be at least 60-90 minutes. The field note method will be used to document nonverbal information such as date, time, location, and the process description of the interview.

### Analysis Plan

Once the data have been gathered from the participants, it will be organized and prepared for analysis and then typed and sorted into different categories. The data will be read and its meaning reflected upon to thoroughly to understand the participants’ responses. In this process, we will also write several notes regarding the data. The second step will be to conduct deeper analysis through data coding, which includes processing material or information into text, extracting, and sorting them into several categories. These categories will be labeled with specific terms based on the participants’ responses. The categories and themes will then be analyzed to narrow them down to a few themes or categories. These themes will be the main result of the qualitative research, which will later create a more complex analysis, becoming a general description. The third step will involve describing the themes and representing them in narratives or qualitative reports. This narrative covers the explanation about event chronology, certain themes (along with the subthemes, specific illustrations, perspectives, and quotations), or interconnections between themes. Lastly, we will interpret the data based on the questions given to the participants and their responses to gain the essence of their ideas. This interpretation will later become the meaning that comes from the participants’ experience, which aligns with the aim of this study—discovering the participants’ meaning of life. This interpretation will not be our conclusion but rather the participants’ ideas in their own words. Upon the completion of the analysis, the data will be presented in charts, narratives, and recorded quotations from the interview.

### Ethics Approval

Approval for the study’s ethics was received from the Medical and Health Research Ethics Committee, Faculty of Medicine, Public Health and Nursing, Universitas Gadjah Mada, Yogyakarta, Indonesia (KE/FK/0747/EC/2021).

## Results

This study received a grant in May 2021 and gained permission from the Medical and Health Research Ethics Committee of Universitas Gadjah Mada, Indonesia, on July 1, 2021. Data collection started on August 12, 2021, and the results are expected to be published in 2022.

## Discussion

### Expected Findings

This study aims to examine the experiences and needs of patients with T2DM in treating the disease. The data in this study will be analyzed using SCT, which involves personal factors, behavior, and environment. According to the SCT, the details gathered from the patients should include knowledge, self-efficacy, outcome expectancy, outcome experience, worry, illness belief, treatment belief, diet, physical activity, medicine intake, treatment pattern, support system, as well as ethnic and cultural influences. The study results will be used to create and develop an educational intervention model tested on patients with T2DM. Hopefully, with a proper educational intervention model, the medication knowledge, attitude, adherence, and hemoglobin A_1c_ levels of patients with T2DM can be improved.

### Conclusions

The results of this study will be used to design an educational intervention model suitable for patients with T2DM to improve their knowledge, attitude, and adherence.

## Abbreviations

SCT: Social Cognitive Theory
T2DM: type 2 diabetes mellitus
